# Supplementary material for: Radiotherapy in the Management of Gastrointestinal Stromal Tumors: A Systematic Review
Source: Cancers (Basel). 2022 Jun 28;14(13):3169. doi: 10.3390/cancers14133169 (PMC9265110; doi:10.3390/cancers14133169)
Supplement: Supplementary file 1 [file cancers-14-03169-s001.zip › Table S2.pdf]

Table S2. Methodological quality of included studies (adapted from the Joanna Briggs Institute (JBI) Critical Appraisal Checklist for Case Reports and Case series)

Checklist questions	Shioyama et al.2001[50]	Pollock et al.2001[25]	Akiyama et al.2004[37]	Puri et al.2006[44]	Boruban et al.2007[23]	Barrière et al.2009[41]	Ciresa et al.2009[52]	Hamada et al.2010[27]	Tezcan et al.2011[30]	Knowlton et al.2011[16]	Naoe et al.2011[19]	Lolli et al.2011[10]	Di Scioscio et al.2011[29]	Abuzakhm et al.2011[34]
1. Were patient's demographic characteristics clearly described?	Yes	Yes	Yes	Yes	Yes	Yes	Yes	Yes	Yes	Yes	Yes	Yes	Yes	Yes
2. Was the patient's history clearly described and presented as a timeline?	Yes	No	Yes	Yes	Yes	No	No	Yes	Yes	No	Yes	Yes	Yes	Yes
3. Was the current clinical condition of the patient on presentation clearly described?	No	Yes	Yes	Yes	Yes	Yes	Yes	No	No	Yes	Yes	No	Yes	Yes
4. Were diagnostic tests or assessment methods and the results clearly described?	Yes	Yes	Yes	Yes	Yes	Yes	Yes	Yes	Yes	No	Yes	Yes	Yes	Yes
5. Was the intervention(s) or treatment procedure(s) clearly described?	Yes	Yes	Yes	No	Yes	Yes	Yes	No	Yes	Yes	No	Yes	No	No
6. Was the post-intervention clinical condition clearly described?	Yes	Yes	Yes	No	Yes	Yes	Yes	No	Yes	No	No	Yes	No	No
7. Were adverse events (harms) or unanticipated events identified and described?	No	Yes	No	No	No	No	Yes	No	No	Yes	No	No	No	No
8. Does the case report provide takeaway lessons?	Yes	Yes	Yes	Yes	Yes	Yes	Yes	Yes	Yes	Yes	Yes	Yes	Yes	Yes
9. Was the case report presented according to CARE checklist?	No	No	No	No	No	No	No	No	No	No	No	No	No	No
Methodological quality*	Moderate	Moderate	Moderate	Low	Moderate	Moderate	Moderate	Low	Moderate	Low	Low	Moderate	Low	Low

Checklist questions	Wong et al.2011[38]	Slimack et al.2012[36]	Halpern et al.2012[28]	Feki et al.2012[35]	Drazin et al.2013[39]	Takeuchi et al.2014[26]	Sato et al.2014[40]	Aktan et al.2015[31]	Gupta et al.2016[24]	Gatto et al.2017[22]	Loaiza-Bonilla et al.2017[46]	Badri et al.2018[42]	Jang et al.2018[43]	Yang et al.2018[51]
1. Were patient's demographic characteristics clearly described?	Yes	Yes	Yes	Yes	Yes	Yes	Yes	Yes	Yes	Yes	Yes	Yes	Yes	Yes
2. Was the patient's history clearly described and presented as a timeline?	No	Yes	No	Yes	Yes	Yes	No	Yes	Yes	Yes	Yes	No	Yes	Yes
3. Was the current clinical condition of the patient on presentation clearly described?	Yes	Yes	Yes	Yes	Yes	Yes	Yes	Yes	Yes	Yes	No	Yes	Yes	Yes
4. Were diagnostic tests or assessment methods and the results clearly	Yes	Yes	No	Yes	Yes	Yes	Yes	Yes	Yes	Yes	Yes	Yes	Yes	Yes

described?														
5. Was the intervention(s) or treatment procedure(s) clearly described?	No	No	Yes	No	Yes	No	Yes	Yes	Yes	Yes	No	No	Yes	Yes
6. Was the post-intervention clinical condition clearly described?	No	No	Yes	Yes	No	Yes	Yes	Yes	Yes	Yes	Yes	No	No	Yes
7. Were adverse events (harms) or unanticipated events identified and described?	No	No	Yes	No	No	No	No	No	No	Yes	No	No	No	No
8. Does the case report provide takeaway lessons?	Yes	Yes	Yes	Yes	Yes	Yes	Yes	Yes	Yes	Yes	Yes	Yes	Yes	Yes
9. Was the case report presented according to CARE checklist?	No	No	No	No	No	No	No	No	No	No	No	Yes	No	No
Methodological quality*	Low	Low	Low	Low	Low	Low	Moderate	Moderate	Moderate	Moderate	Low	Low	Low	Moderate

Checklist questions	Katayanagi et al.2019[32]	Yilmaz et al.2020[17]	Carvalho et al.2020[18]	Andruska et al.2020[21]	Lo et al.2020[33]	Maria et al.2022[20]	Al-Jarani et al.2022[54]	Checklist questions	Baik et al.2007[45]	Cuaron et al.2013[11]	Joensuu et al.2015[47]	Rathmann et al.2015[49]
1. Were patient's demographic characteristics clearly described?	Yes	Yes	Yes	Yes	Yes	Yes	Yes	1. Were there clear criteria for case inclusion?	No	No	Yes	No
2. Was the patient's history clearly described and presented as a timeline?	Yes	Yes	Yes	Yes	Yes	Yes	No	2. Were standard, credible methods used to identify disease or health problems?	Yes	No	Yes	Yes
3. Was the current clinical condition of the patient on presentation clearly described?	No	Yes	Yes	Yes	Yes	Yes	Yes	3. Were effective methods used to diagnose disease or health problems?	Yes	No	Yes	Yes
4. Were diagnostic tests or assessment methods and the results clearly described?	Yes	Yes	Yes	Yes	Yes	Yes	No	4. Was the inclusion of patients consistent?	Yes	Yes	No	Yes
								5. Was the inclusion of patients comprehensive?	No	No	No	No
5. Was the intervention(s) or treatment procedure(s) clearly described?	Yes	Yes	No	Yes	Yes	Yes	No	6. Were patient's demographic characteristics clearly described?	Yes	Yes	Yes	Yes
6. Was the post-intervention clinical condition clearly described?	No	Yes	No	No	No	Yes	No	7. Was the clinical information of patients clearly described?	Yes	Yes	Yes	Yes
7. Were adverse events (harms) or unanticipated events identified and described?	No	Yes	No	No	No	Yes	Yes	8. Were outcomes or follow-up results clearly reported?	Yes	Yes	Yes	Yes
								9. Was geographical/sociological information of patients clearly reported?	No	No	No	No
8. Does the case report provide takeaway lessons?	Yes	Yes	Yes	Yes	Yes	Yes	Yes					
9. Was the case report presented according to CARE checklist?	No	No	No	No	No	No	No	10. Were statistical analysis methods appropriate?	No	Yes	Yes	Yes
Methodological quality*	Low	Moderate	Low	Low	Low	Moderate	Low	Methodological quality*	Low	Low	Low	Low

Checklist questions	Omari et al.2019[48]	Patterson et al.2022[53]

1. Were there clear criteria for case inclusion?	Yes	No
2. Were standard, credible methods used to identify disease or health problems?	Yes	No
3. Were effective methods used to diagnose disease or health problems?	Yes	No
4. Was the inclusion of patients consistent?	Yes	Yes
5. Was the inclusion of patients comprehensive?	No	No
6. Were patient's demographic characteristics clearly described?	Yes	Yes
7. Was the clinical information of patients clearly described?	Yes	Yes
8. Were outcomes or follow-up results clearly reported?	Yes	Yes
9. Was geographical/sociological information of patients clearly reported?	No	Yes
10. Were statistical analysis methods appropriate?	Yes	Yes
Methodological quality*	Moderate	Low
